# TGEV infection up-regulates FcRn expression via activation of NF-κB signaling

**DOI:** 10.1038/srep32154

**Published:** 2016-08-24

**Authors:** Jinyue Guo, Fei Li, Shaoju Qian, Dingren Bi, Qigai He, Hui Jin, Rui Luo, Shaowen Li, Xianrong Meng, Zili Li

**Affiliations:** 1 College of Veterinary Medicine, Huazhong Agricultural University, Wuhan 430070, China; 2 State Key Laboratory of Agricultural Microbiology, Huazhong Agricultural University, Wuhan 430070, China

## Abstract

It has been well characterized that the neonatal Fc receptor (FcRn) transports maternal IgG to a fetus or newborn and protects IgG from degradation. We previously reported that FcRn is expressed in a model of normal porcine intestinal epithelial cells (IPEC-J2). Transmissible gastroenteritis is an acute enteric disease of swine that is caused by transmissible gastroenteritis virus (TGEV). How porcine FcRn (pFcRn) expression is regulated by pathogenic infection remains unknown. Our research shows that IPEC-J2 cells infected with TGEV had up-regulated pFcRn expression. In addition, the NF-κB signaling pathway was activated in IPEC-J2 cells by TGEV infection. Furthermore, treatment of TGEV-infected IPEC-J2 cells with the NF-κB-specific inhibitor BAY 11-7082 resulted in down-regulation of pFcRn expression. Transient transfection of pFcRn promoter luciferase report plasmids with overexpression of NF-κB p65 transcription factor enhanced the activation of the luciferase report plasmids. We identified four NF-κB transcription factor binding sites in the promoter region of this gene using luciferase reporter system, chromatin immunoprecipitation, electromobility shift assay, and supershift analysis. Together, the data provide the first evidence that TGEV infection up-regulates pFcRn expression via activation of NF-κB signaling.

Immunoglobulin G is a major Ig isotype in mucosal secretions and is involved in host defense. It has now been 50 years since the remarkable foresight by F.W.R. Brambell, who described a saturable receptor that transports maternal IgG to a fetus or newborn. A few years later, he put forth the hypothesis of the presence of a similar receptor that protected IgG from degradation, eventually identified as the neonatal Fc receptor (FcRn)^1^. FcRn was originally isolated from the intestine of neonatal rodents and identified as the receptor responsible for the transmission of maternal antibodies from mother to pup^2^,^3^,^4^,^5^. In recent decades, researchers have showed that FcRn is most closely structurally related to the major histocompatibility complex class I molecule and is composed of a heavy chain associated noncovalently with β2-microglobulin (β2m)^5^,^6^. FcRn was also shown to bind IgG at the CH2-CH3 interface in a pH-dependent way. Binding occurs in acidic (pH ≤ 6.5) environments, and IgG is released at neutral (pH ≥ 7.4) conditions^7^,^8^. FcRn is a transport receptor which mediated transfer of IgGs across the human placental barrier or the rodents intestinal epithelial barrier to a fetus or newborn^5^,^9^,^10^. FcRn, therefore, plays a major role in the passive acquisition of maternal immunity by newborn mammals. In addition, FcRn is capable of protecting IgG from degradation and maintaining IgG levels in the bloodstream^11^.

FcRn has been indicated to be expressed in a variety of mammalian species, including mouse, human, rat, sheep, cow, pig, possum and camel^12^. The level of FcRn expression plays an important role in controlling IgG levels in tissues and blood^13^. Some studies have shown that mice deficient in either β2m or the heavy chain of FcRn fail to transport IgG, meanwhile the serum half-life of IgG is shortened^14^,^15^. More recently, several publications indicated that transgenic (Tg) mice that over-express bovine FcRn in the mammary gland have increased IgG levels in both milk and serum^16^. Meanwhile, some researchers have reported FcRn overexpression by Tg modification in mice and rabbits not only prolongs the IgG half-life but also enhances the humoral immune response of these animals^17^,^18^,^19^. More specifically, these Tg animals displayed significantly larger spleens containing a higher number of Ag-specific B cells and plasma cells in response to immunization, increased antibody diversity and prolonged Ag-specific IgG half-life^20^. This augmented immune response is also reflected in the ability of FcRn Tg mice to produce high levels of Ag-specific antibodies, B cells and plasma cells to weakly immunogenic targets or evade recognition by the immune system^21^.

Nuclear factor-κB (NF-κB) is a family of transcription factors that mediates signal-induced expression of numerous genes involved in the innate and adaptive immune responses, inflammation, and autoimmune diseases^22^. Some articles have reported that NF-κB signaling regulates functional expression and function of the human and bovine FcRn^23^,^24^. In the present study, we have analyzed the NF-κB binding site in the promoter of pFcRn gene.

Transmissible gastroenteritis virus (TGEV) is a member of the family Coronaviridae in the order Nidovirales^25^. It replicates in the differentiated enterocytes covering the villi of the porcine small intestine and causes severe gastroenteritis in young TGEV-seronegative pigs. Diseased pigs often present with vomiting, dehydration, and severe diarrhea. Consistent with *in vivo* pathological changes, TGEV infection induces morphological and biochemical changes in host cells and some porcine cell lines *in vitro*. Previous studies have reported that TGEV infection induces apoptosis in PK-15 cells via activation of the p53 signaling pathway^26^. Quantitative proteomic analysis reveals that TGEV infection activates the janus kinase signal transducer and activator of the transcription 1 (JAK-STAT1) signaling pathway^27^. Several publications have also shown that TGEV could infect IPEC-J2 cells, and that it could also down-regulate proteins involved in tight and adherens junctions to alter the epithelial barrier integrity^28^. These findings suggest that TGEV infection induces a number of changes in host-cell biology that could influence cells function.

In the present study, we investigated how TGEV infection activated the NF-κB pathway *in vitro* and up-regulated pFcRn expression in IPEC-J2 cells. Our studies showed that pFcRn expression can be triggered by TGEV infection, through NF-κB signal activation. An NF-κB-specific inhibitor significantly down-regulated expression of pFcRn gene by TGEV infection. Furthermore, we identified the direct involvement of NF-κB-specific binding sites by using several complementary strategies. These observations support the hypothesis that up-regulation of pFcRn expression following virus infection appears to be an innate immune response against invading pathogens that could help the host clear infection effectively.

## Results

### TGEV up-regulates pFcRn mRNA and protein in IPEC-J2 cells

**Figure 1:**
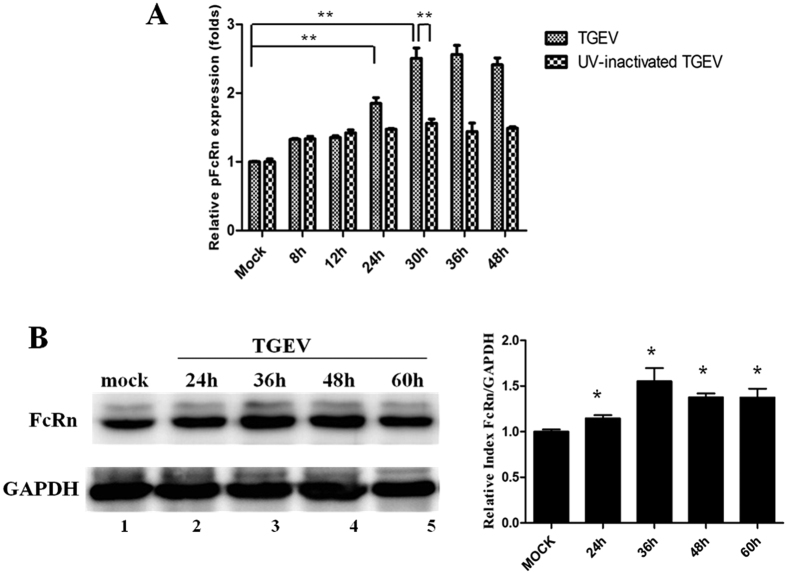
pFcRn expression in response to TGEV infection. (**A**) Quantitative real-time RT-PCR analysis of pFcRn mRNA in IPEC-J2 cells by TGEV infection (MOI = 1) for the indicated times (8, 12, 24, 30, 36 and 48 h). (**B**) Western blot. Cell lysates from IPEC-J2 (lane 1) and TGEV-infected IPEC-J2 (lanes 2–5) were separated by electrophoresis in a 12% SDS-polyacrylamide gel, transferred to a PVDF membrane, and blotted with an pFcRn-specific (top panel) or a GAPDH-specific Ab (bottom panel). Blots were then incubated with a HRP-conjugated secondary Ab and visualized using chemiluminescence. The bar graphs represented results of three independent experiments. Intensities of proteins bands were calculated from the peak area of densitograms by using image software.

To determine whether TGEV is capable of modulating pFcRn expression in IPEC-J2 cells, infectious TGEV or UV-inactivated virus was adsorbed to confluent monolayers of IPEC-J2 cells at an MOI of 1 and pFcRn mRNA was measured 8, 12, 24, 30, 36 and 48 h post-virus exposure by real-time RT-PCR. We found that the level of pFcRn mRNA expression was 2-fold higher at 30, 36 and 48 h post-infection, compared to mock-infected cells (Fig. 1A). However UV-inactivated virus treatment only caused a 1.4-fold up-regulation in pFcRn expression, compared to mock-infected cells (Fig. 1A). Replicating virus had a more pronounced effect than UV-inactivated virus.

To determine whether the increased mRNA levels resulted in increased pFcRn protein production, IPEC-J2 cells were cultured in the presence or absence of infectious virus at an MOI of 1 and pFcRn protein was measured in cell lysates by Western blot (Fig. 1B). Increased levels of pFcRn were detected by 36 h and 48 h TGEV exposure (Fig. 1B). Thus, the elevation in protein levels correlated with the increased mRNA levels by 30 h.

### TGEV infection activates NF-κB signaling pathway

**Figure 2:**
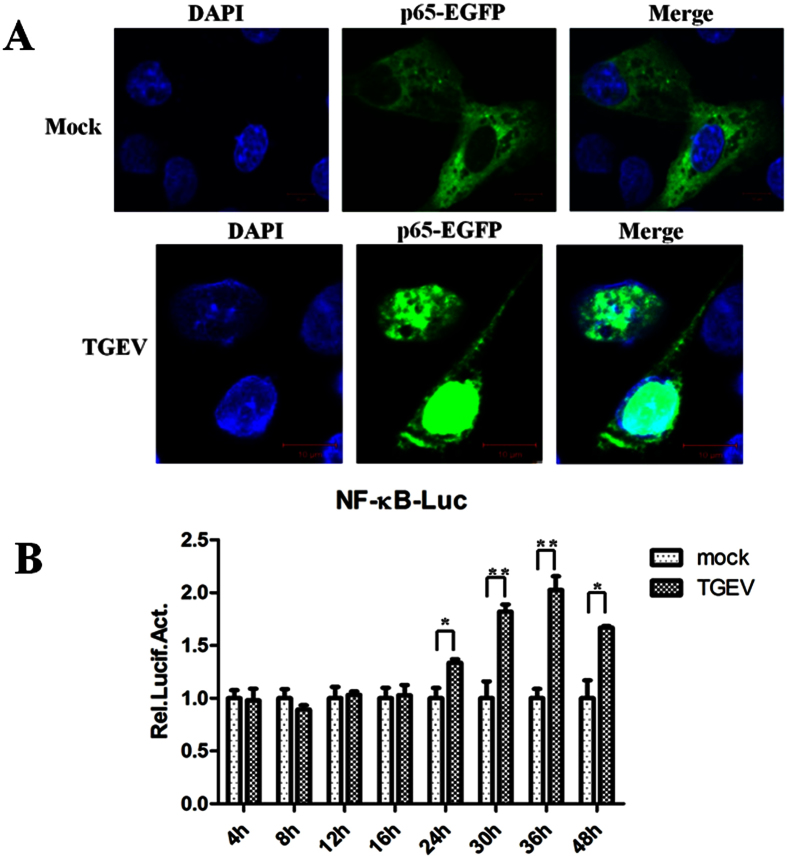
TGEV infection activates NF-κB signaling pathway. (**A**) Nuclear translocation of NF-κB induced by TGEV infection was detected by confocal laser microscopy. Fluorescence was examined by using a Zeiss LSM 510 Meta confocal laser microscope. Scale bar equals 10 μm. (**B**) TGEV enhanced NF-κB-regulated gene expression in time-dependent manner. IPEC-J2 cells transfected with pNF-κB-Luc and pRL-TK plasmids were inoculated with TGEV at 4, 8, 12, 16, 24, 30, 36 and 48 h respectively. Values are mean ± SEM of three independent experiments.

IPEC-J2 cells were transiently transfected with p65-EGFP fusion expression plasmid and the subcellular localization of the fusion protein was analyzed using confocal laser microscopy. As shown in Fig. 2A, p65-EGFP was located exclusively in the cytoplasm in mock-infected IPEC-J2 cells (top panels), but it rapidly translocated to the nucleus when those cells were infected with TGEV (bottom panels).

Cells were cotransfected with 450 ng/well of NF-κB luciferase reporter plasmid pNF-κB-Luc and 50 ng/well of the Renilla luciferase construct pRL-TK which was served as an internal control. Twenty-four hours later, cells were infected or control-infected with TGEV. Cells were harvested at the indicated times and luciferase activity was measured using a dual-luciferase Assay System. Data represent relative firefly luciferase activity normalized to Renilla luciferase activity. NF-κB-regulated luciferase expression was significantly enhanced during TGEV infection at 24, 30, 36 and 48 h, compared to mock-infected samples (Fig. 2B).

These two results demonstrated that the classical pathway of NF-κB was activated in IPEC-J2 cells by TGEV infection.

### Effect of NF-κB inhibition on pFcRn expression

**Figure 3:**
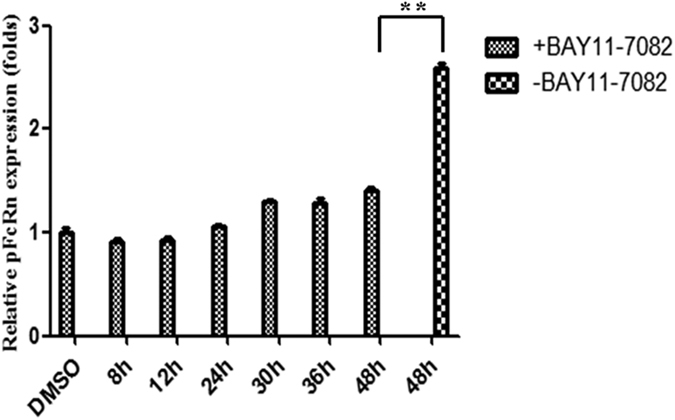
Effect of NF-κB inhibitors on the expression of pFcRn. IPEC-J2 cells were incubated with or without the NF-κB -specific inhibitor BAY 11-7082 (10 μM) for 30 min. IPEC-J2 cells were subsequently infected with or without TGEV. At the end of the incubation periods for the indicated times, RNA was isolated and analyzed by quantitative real-time RT-PCR.

Inhibition of pFcRn induction by BAY 11-7082, a small molecule that blocks the phosphorylation of IκBα, suggested that the early proinflammatory response involved activation of the classical NF-κB pathway. IPEC-J2 cells were pretreated with BAY 11-7082 (10 μM) for 30 min and subsequently infected by TGEV. Treatment with BAY 11-7082 significantly reduced TGEV infected pFcRn mRNA levels to that of the TGEV infected cells without BAY 11-7082 treatment as assessed by real-time RT-PCR at 48 h (Fig. 3). Therefore inhibition of pFcRn induction by BAY 11-7082 suggested that early activation of the classical pathway of NF-κB activation was critical for subsequent up-regulation of pFcRn mRNA.

### Construction of pFcRn promoter luciferase report plasmids

Neural Network Promoter Prediction software was used for searching putative transcription start sites in the 5′-flanking region of the pFcRn gene (minimum promoter score: 0.8). Computational analysis revealed that the pFcRn gene contains two transcription start sites (Fig. S1). Transcription start site-2 has a higher score than the transcription start site-1. Furthermore, the alignment of pFcRn and human FcRn (hFcRn) upstream sequences of the start codon showed that regions near transcription start site-2 of pFcRn gene have a high homology with the transcription start site nucleotide sequence of hFcRn gene (Fig. S2). In the study, the transcription start site-2 was considered as the transcription start site of pFcRn gene (Fig. S1).

To study whether NF-κB regulates pFcRn expression through a mechanism that involves direct binding to a putative regulatory NF-κB binding sequence located in the pFcRn gene, we utilized luciferase reporter constructs of the pFcRn promoter that contained NF-κB p65 binding sites.

**Figure 4:**
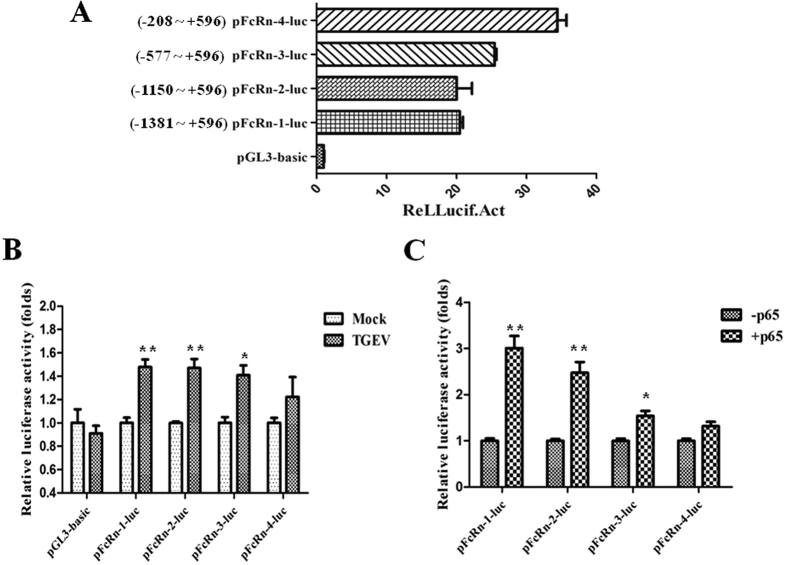
Construction of pFcRn promoter luciferase report plasmids. (**A**) The basal promoter activity of the pFcRn-(1–4)-luc in IPEC-J2 cells. (**B**) TGEV infection up-regulated the activity of pFcRn-(1–3)-luc. IPEC-J2 cells were transiently transfected with the pFcRn luciferase reporter plasmids. Twenty-four hours after transfection, cells were either mock infected or TGEV infected. After 36 h of infection, cells were harvested and protein extracts were prepared for luciferase assay as described above. (**C**) Luciferase activity of the pFcRn-(1–4)-luc with overexpression of p65 in IPEC-J2 cells. IPEC-J2 cells were transiently transfected pFcRn-(1–4)-luc with the p65-Tag2B plasmid together. Luciferase activity was measured 36 h post-transfection. IPEC-J2 cells were harvested and protein extracts were prepared for the luciferase assay as described above.

We created four luciferase reporter gene constructs with sequentially shortened fragments of the promoter region: pFcRn-luc-1 (−1381~+596), pFcRn-luc-2 (−1150~+596), pFcRn-luc-3 (−577~+596) and pFcRn-luc-4 (−208~+596). Furthermore, transient transfection of the four luciferase reporter plasmids in IPEC-J2 cells revealed that these plasmids significantly increased the basal promoter activity above that of the empty vector (Fig. 4A). Next, we tested whether pFcRn promoter activity could be up-regulated by TGEV infection. The results showed that luciferase activity was significantly enhanced using the pFcRn-luc-1, pFcRn-luc-2 and pFcRn-luc-3 constructs during TGEV infection compared with mock-infected IPEC-J2 cells, while the luciferase reporter construct harboring the shortest fragment of pFcRn-luc-4 had not significant differences comparing with mock-infected IPEC-J2 cells (Fig. 4B).

To directly assess the involvement of NF-κB in pFcRn expression, we investigated whether overexpression of NF-κB p65 affects the promoter activity of pFcRn. We transiently transfected IPEC-J2 cells with the p65-Tag2B plasmid. We found that pFcRn-luc-1, pFcRn-luc-2 and pFcRn-luc-3 constructs were induced in the presence of p65, while pFcRn-luc-4 had not significant differences comparing with mock group (Fig. 4C). These results indicate that the overexpression of NF-κB p65 is sufficient to up-regulate pFcRn expression. Meanwhile the NF-κB sensitive region is located in the sequence between −1381 and −208 of the pFcRn promoter.

### Screening for NF-κB binding sites adjacent to the pFcRn gene

**Figure 5:**
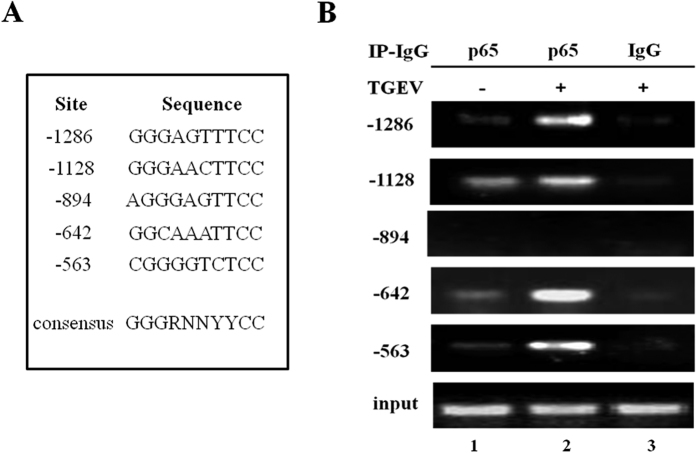
Screening of NF-κB binding sites in the pFcRn gene by ChIP. (**A**) The putative NF-κB binding sites in the pFcRn gene are listed. Numbers represent the putative NF-κB binding sites relevant to the transcription start site of the pFcRn gene. In the consensus NF-κB sequence, R is an A or G purine, N is any nucleotide and Y is C or T. (**B**) NF-κB p65 components are present at pFcRn promoter *in vivo* in TGEV infected cells, (MOI = 1) for 30 h. ChIP assays were performed using p65-specific Abs (lane 2). IgG was used as a negative control (lane 3). Purified DNA fragments were subjected to PCR analysis using primer pairs (Table 1). ChIP was performed at least three times.

The canonical NF-κB DNA binding sequence is a common 10-bp consensus DNA element that has been identified as 5′-GGGRNNYYCC-3′ (where R is an A or G; N is any nucleotide; Y is C or T)^22^. We searched for putative NF-κB-binding sequence(s) along this promoter region (−1381~−208). Computational inspection revealed that the promoter of the pFcRn gene contained sequences with a similarity to the NF-κB consensus sequence (Fig. 5A). We used a ChIP assay to verify that these putative NF-κB binding sequences had the capability to directly bind NF-κB proteins in living cells. First, cross-linking the DNA with bound NF-κB proteins *in situ* in TGEV infection verus mock-infected IPEC-J2 cells, NF-κB-DNA complexes were co-incubated with p65 specific antibody. Then, the purified DNA fragments were measured by PCR. As shown in Fig. 5B, PCR with specific primers (Table 1) produced a band from DNA coprecipitated with p65 (Fig. 5B, −1286, −1128, −642 and −563), however the sequence (−894) failed to produced a band. In a negative control, immunoprecipitation with normal mouse IgG did not generate any corresponding PCR products (Fig. 5B, lane 3). The results suggest that p65 transcription factor interacted with the four NF-κB binding sequences of pFcRn gene in IPEC-J2 cells.

### EMSA and supershift assays confirmed the four NF-κB binding sites in the pFcRn promoter

**Figure 6:**
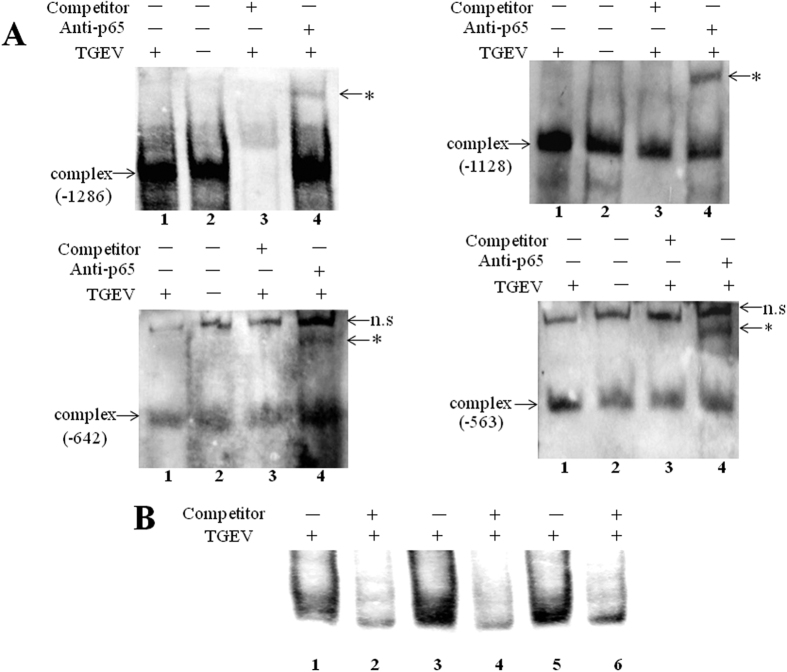
EMSA and supershift assays confirmed four NF-κB binding sites in the pFcRn promoter. (**A**) Nuclear extracts prepared from non-infected and TGEV-infected IPEC-J2 were incubated with biotin-labeled probe. Biotin-labeled probes representing the NF-κB binding sites (−1286, −1128, −642, −563) in the absence or presence p65-specific Abs. Distinct κB-specific protein-DNA complexes (lower arrow, κB-specific complex) were detected using nuclear extracts (lanes 1 and 2), and these complexes could be further shifted by p65-specific Abs (*supershifted κB-specific complex, lane 4). n.s, non-specific bands. (**B**) a competition assay was performed. Nonlabeled oligonucleotide in −1128, −642 and −563 NF-κB binding sequences was added to a 100-fold excess (Fig. 6B, lanes 2, 4 and 6). Distinct κB-specific protein-DNA complexes were detected using nuclear extracts (Fig. 6B, lanes 1, 3 and 5).

EMSA and supershift assays further confirmed the four NF-κB binding sites that were identified from the ChIP assay. Nuclear extracts co-incubated with oligonucleotides containing NF-κB binding sequences (Fig. 6A, −1286, −1128, −642 and −563). As shown in Fig. 6A, a higher amount of complexes formed when bound to nuclear extracts from TGEV-infected cells than when bound to nuclear extracts from mock-infected cells. These complexes were further shifted by anti-p65 monoclonal antibody, indicating that the complexes contained p65 transcription factor (Fig. 6A, upper arrow, supershifted NF-κB-specific complex, lane 4). To verify the binding specificity, a competition assay was performed. Although the specific band could not be significantly inhibited by unlabeled oligonucleotides in −1128, −642 and −563 NF-κB binding sequences (Fig. 6A, lane 3). Nonlabeled oligonucleotide was added to a 100-fold excess, the specific band was inhibited by unlabeled oligonucleotides in −1128, −642 and −563 NF-κB binding sequences (Fig. 6B, lanes 2, 4, 6).

## Discussion

FcRn has more recently been shown to express in a variety of mammalian species^12^. Meanwhile, several studies have reported the distribution, function, and regulation of human and rodent FcRn expression^9^,^24^,^29^,^30^. Our study demonstrated that pFcRn mediated bidirectional IgG transport across polarized IPEC-J2 cells that potentially provide the mucosal protection^31^. Some studies have showed that up-regulation of FcRn expression can augment the IgG transport across the polarized epithelial cells^24^. FcRn overexpression boosts humoral immune response in transgenic mice^20^.

TGEV is an enveloped, non-segmented, single-stranded positive-sense RNA virus^32^. The 3′ one-third of the genome encodes viral structural and accessory proteins, including the spike (S) glycoprotein, the membrane (M) glycoprotein, the small envelope (E) glycoprotein, the nucleocapsid (N) protein, and three accessory proteins 3a, 3b, and 7, meanwhile 5′-proximal two-thirds of the genome encode the viral replicase^33^. Our study is the first to show that TGEV infection up-regulates pFcRn mRNA and protein in IPEC-J2 cells (Fig. 1). Levels of pFcRn mRNA in UV-inactivated virus treatments are only 1.4-fold lower compared to replicating virus infection. These results show that TGEV-mediated FcRn up-regulation is mainly related to the replication of virus, meanwhile UV-inactivated TGEV-mediated FcRn up-regulation maybe related to viral structural and accessory proteins.

Some researchers have shown that FasL- and mitochondria-mediated pathways were activated by TGEV infection, which induces apoptosis in PK-15 cells^34^. Moreover, TGEV infection promoted the activation of p53 signaling to induce cell apoptosis^35^. Our results show that TGEV infection activates the NF-κB signaling pathway, determined by a p65 nuclear translocation assay and NF-κB luciferase report system assay (Fig. 2). Furthermore, pFcRn expression induced by TGEV infection was strongly reduced by the NF-κB-specific inhibitor BAY 11-7082 in IPEC-J2 cells (Fig. 3). This was corroborated by the overexpression of NF-κB p65, which up-regulated the activation of pFcRn promoter luciferase report plasmids. This complementary experiment lessens the concern of specificity or toxicity of the chemical inhibitor.

The promoter regions for human and bovine FcRn have been analyzed, and the regulation of expression has been shown to be mediated with p65 transcription factors^23^,^24^. In this study, we analyzed the 5′-flanking region of the pFcRn gene by TESS and TFSEARCH programs, and found there are five potential NF-κB transcription factor p65 binding sites between position −1381 and −208. Therefore, we constructed luciferase reporter plasmids with sequentially shortened fragments of the FcRn promoter region. Transient transfection of the pFcRn promoter luciferase reporter plasmids revealed that pFcRn-luc-(1–3) plasmids resulted in increased promoter activity in the presence of TGEV infection (Fig. 4B), further demonstrating that TGEV up-regulates pFcRn expression in IPEC-J2 cells. pFcRn-luc-(1–3) plasmids were also activated by overexpression of NF-κB p65 plasmid (Fig. 4C). These results showed that the NF-κB sensitive region of FcRn promoter is located in the sequence between −1381 and −208.

We mapped four NF-κB binding sequences in our ChIP experiment (Fig. 5). This result was further confirmed by EMSA and supershift analysis (Fig. 6). These results indicated strong and effective molecular interactions between NF-κB p65 and the selected transcription binding sites of the pFcRn promoter.

NF-κB is a ubiquitous transcription factor that regulates the transcription of genes such as chemokines, cytokines, proinflammatory enzymes, adhesion molecules, proinflammatory transcription factors and so on. Based on previous studies, NF-κB is activated via two distinct kinase-dependent pathways, the classical NF-κB pathway and the alternative NF-κB pathway. While our research mainly concentrated on the classical NF-κB pathway, its activity can be modulated significantly by additional factors that transfer into the nucleus and bind to NF-κB binding sequences. In this regard, we analyzed the 5′-flanking region of the pFcRn gene. and found there are several other transcriptional factors in this region, such as AP-1 and Sp1. Some studies have suggested that NF-*κ*B dimers can act synergistically with AP-1 and Sp1 transcriptional factors to influence gene regulation^36^,^37^,^38^. These protein-protein interactions may be involved in mediating transcriptional regulation of the pFcRn gene in response to stimuli and can functionally cooperate to elicit maximal activation of the promoter.

In summary, TGEV infection up-regulates pFcRn expression in IPEC-J2 cells, and activates the NF-κB signaling pathway. Furthermore, pFcRn expression was strongly reduced by inhibitor BAY 11-7082 treatment after TGEV infection. This result deduced that the up-regulation of FcRn expression by TGEV infection may be related to NF-κB signaling pathway. We constructed FcRn promoter luciferase report plasmids by computational inspection. According to the analysis of the activation of pFcRn promoter luciferase report plasmids by TGEV infection and overexpression p65 plasmid, the NF-κB sensitive region of FcRn promoter is located in the sequence between −1381 and −208. There are four NF-κB binding sites confirmed by ChIP and EMSA experiments. In the study, upregulation of pFcRn expression following virus infection may play an important role in effectively protecting the mucosal surface from the pathogens invasion.

## Materials and Methods

### Cell lines, antibodies, virus and plasmids

IPEC-J2 cells were cultured in Dulbecco’s Modified Eagle’s Media (DMEM)/Ham’s F-12 (1:1) (Hyclone, USA) with 10% fetal bovine serum (Gibco, USA) and 1% penicillin/streptomycin. All cells were grown in a humidified atmosphere of 5% CO_2_ at 37 °C. TGEV strain WH-1 (GenBank accession no. HQ462571), which was isolated in China, was propagated in PK-15 cells.

Affinity-purified rabbit anti-cytoplasmic tail of porcine FcRn (anti-pFcRn-CT) polyclonal antibody was prepared in our laboratory. Horseradish peroxidase (HRP)-conjugated goat anti-rabbit and rabbit anti-mouse IgG were purchased from Thermo Scientific Pierce (USA). Mouse anti-GAPDH monoclonal antibody was purchased from BOSTER (China).

p65-EGFP fusion expression plasmid was prepared in our laboratory, p65 gene was PCR subcloned into pEGFP-C2 vector. p65-Tag2B plasmid encoding p65 was prepared in our laboratory, p65 gene was PCR subcloned into pCMV-Tag 2B vector.

### Quantitative real-time RT-PCR

Confluent monolayers of IPEC-J2 cells were inoculated with TGEV at a multiplicity of infection (MOI) of 1 for 8, 12, 24, 30, 36, and 48 h at 37 °C. Total RNA was extracted from IPEC-J2 cells with TRIzol Reagent (Invitrogen, USA). Then, total RNA was reverse-transcribed into cDNA using reverse transcriptase (TaKaRa, China). Real-time RT-PCR was performed in triplicate in three separate experiments using pFcRn primers (5′-GGCGACGAGCACCACTACTG-3′ and 5′-AGCCGACCATGATTCCAACC-3′) and GAPDH primers (5′-ACATGGCCTCCAAGGAGTAAGA-3′ and 5′-GATCGAGTTGGGGCTGTGACT-3′) and the SYBR Premix Ex Taq II (TaKaRa, China). All reactions were performed for 40 cycles: 5s at 95 °C and 30s at 60 °C. pFcRn expression was calculated following normalization to GAPDH levels by the comparative delta delta threshold cycle (ΔΔC_T_) method. The specific amplification reactions were confirmed by melt curve analysis.

### Western blotting

Lysates from IPEC-J2 cells were prepared as described previously^39^. The total proteins were resolved on a 12% SDS-polyacrylamide gels under reducing conditions and electrotransferred onto a polyvinylidene fluoride membrane (Bio-Rad, USA). Affinity-purified rabbit anti-pFcRn-CT polyclonal antibody and mouse anti-GAPDH monoclonal antibody were used as primary antibodies, then HRP-conjugated goat anti-rabbit IgG or HRP-conjugated rabbit anti-mouse IgG were used as secondary antibodies. Western blot analysis was performed as described previously^31^.

### Confocal laser microscope assay

IPEC-J2 cells were grown to approximately 60–70% confluency on coverslips placed in 24-well plates. After transfection with 1.0 μg of p65-EGFP fusion expression plasmid per well, cells were mock-infected or infected with TGEV at a MOI = 1. 36 h after stimulation, cells were fixed with 4% paraformaldehyde, permeabilized with 0.1% Triton X-100, and stained with DAPI (Invitrogen, USA) to detect nuclei.

### Construction of reporter plasmids

Four fragments of the 5′-flanking region of the pFcRn were PCR subcloned into the luciferase expression vector pGL3 (Promega, USA) through Sac I and Hind III digestion. pFcRn-luc-1, containing sequences from −1381 to +596 of the pFcRn gene promoter was amplified by the PCR primer pairs (5′-GCGAGCTCGCTATAG CTCTGATTCGACC-3′ and 5′-CAAGCTTCTGAGCGGGAGACCTGGGG-3′); pFcRn-luc-2, which contains the segment from −1150 to+596 of the pFcRn gene promoter was amplified using the reverse primers described above and a forward primer (5′-GCGAGCTCGCTTCAGCTGGACCCGTAGC-3′). Plasmid pFcRn-luc-3, which contains the segment from −577 to +596 of the pFcRn gene promoter, was generated using the reverse primers described above for PCR amplification and a forward primer (5′-GCGAGCTCTACTTAAAGGGGTACGGGGT-3′). Plasmid pFcRn-luc-4, which contains the segment from −208 to +596 of the pFcRn gene promoter and was generated using the reverse primers described above for PCR amplification and a forward primer (5′-GCGAGCTCGGGGGTGCTGACGAGGTAAGAA-3′).

### Transient transfection and luciferase assay

IPEC-J2 cells were transiently transfected with Lipofectamine 2000 (Invitrogen, USA). Cells were transfected with 950 ng of pFcRn promoter luciferase report plasmids and 50 ng of a Renilla luciferase pRL-TK control plasmid. Twenty-four hours later, cells were infected with or without TGEV (MOI = 1). Cells were harvested at the indicated time and luciferase activity was measured using a dual-luciferase Assay System (Promega, USA). The values for firefly luciferase were normalized to the Renilla luciferase activity and expressed as fold activation over the mock-infected group.

In order to detect whether TGEV infection activates NF-κB signaling pathway, IPEC-J2 cells were cotransfected with 450 ng/well of NF-κB luciferase reporter plasmid pNF-κB-Luc and 50 ng/well of the Renilla luciferase construct pRL-TK. Cells were infected or control-infected with TGEV and were harvested at the indicated times and luciferase activity was measured using a dual-luciferase Assay System.

### Chromatin immunoprecipitation (ChIP)

ChIP was performed according to the manufacturer’s recommendations (Epigentek, USA). In brief, IPEC-J2 cells were infected with or without TGEV (MOI = 1) for 30 h. The cells were fixed with 1% formaldehyde. The nuclei were isolated and DNA was sheared by sonication. Chromatin was immunoprecipitated with 1 μg of Ab specific for p65 or with 1 μg of normal IgG as negative control for 2 h at room temperature by an orbital shaker (50–100 rpm). The DNA samples were amplified by PCR primers (Table 1) in optimized conditions.

### Preparation of nuclear extracts, EMSA and supershift assay

Nuclear extracts were prepared using a nuclear and cytoplasmic extraction kit (CWBIO, China). The single-strand oligonucleotides was labeled with biotin on the 3′ end DNA, annealed to form double-stranded oligonucleotides containing the tested NF-κB sequences from the pFcRn promoter: pFcRn κB-1286 (5′-AAAAATGGGA GTTTCCATTTCCG-3′), pFcRn κB-1128 (5′-GTAGCCTGGGAACTTCCAGAT GCC-3′), pFcRn κB-642 (5′-CCAGAAGAGGCAAATTCCTAGAGAC-3′) and pFcRn κB-563 (5′-AAGGGGTACGGGGTCTCCTTGGGG-3′). For competition assays, a 50-fold excess of nonlabeled oligonucleotide was incubated during the preincubation time. For the supershift assays, 1 μg anti-p65 monoclonal antibody directed against NF-κB p65 was preincubated with the nuclear extracts. The complexes were run on a 5% native polyacrylamide gel. EMSA experiments were performed according to the LightShift chemiluminescent EMSA kit (Thermo Scientific Pierce, USA).

### Statistical analysis

Data from three independent studies were analyzed using ANOVA to identify significant changes between TGEV-infected and mock-infected cells. All results are expressed as mean ± SEM from three independent experiments. P values < 0.05 were considered significant (*P < 0.05 and **P < 0.01).

## Additional Information

**How to cite this article**: Guo, J. *et al.* TGEV infection up-regulates FcRn expression via activation of NF-κB signaling. *Sci. Rep.* **6**, 32154; doi: 10.1038/srep32154 (2016).

## Supplementary Material

Supplementary Information

## Figures and Tables

**Table 1:** PCR primers used in the ChIP assay.

Gene	Forward Primer	Reverse Primer
Co-FcRn-1286	5′-ATGTGCCGTGGGTGTGGCCCTA-3′	5′-AACTTGCATCCCTGATAAGA-3′
Co-FcRn-1128	5′-ATGGCTGTGGTATAGGCTGATA-3′	5′-CCTTTTTACAGCAATACATGCC-3′
Co-FcRn-894	5′-TGGTTGATCCAGACAATAGAAT-3′	5′-GCCGCAGCAGTGATCCCA-3′
Co-FcRn-642	5′-GGCACCATATTGTAGGATTCCA-3′	5′-CAATCACCGATGTGTACGTT-3′
Co-FcRn-563	5′-CATCGGTGATTGCCAGGA-3′	5′-TGCCACCACGGCCACTA-3′
