# Supplementary material for: TGEV infection up-regulates FcRn expression via activation of NF-κB signaling
Source: Sci Rep. 2016 Aug 24;6:32154. doi: 10.1038/srep32154 (PMC4995372; doi:10.1038/srep32154)
Supplement: Supplementary Information [file srep32154-s1.pdf]

TGEV infection up-regulates FcRn expression via activation of NF-κB signaling

Jinyue Guo [a], Fei Li [a], Shaoju Qian[a], Dingren Bi [a, b], Qigai He [a, b], Hui Jin [a, b], Rui Luo[a, b], Shaowen Li [a, b], Xianrong Meng[a, b], Zili Li [a, b, *]

[a] College of Veterinary Medicine, Huazhong Agricultural University, Wuhan 430070, China

[b] State Key Laboratory of Agricultural Microbiology, Huazhong Agricultural University, Wuhan 430070, China

*Corresponding authors.

Tel: +86-27-87288917; Fax: +86-27-87280208;

E-mails: lizili@mail.hzau.edu.cn (Z.L.)

Fig. S1 Analysis of NF-κB transcription factor binding sites in the 5’-flanking region of the pFcRn. The pFcRn-1, pFcRn-2, pFcRn-3 and pFcRn-4 fragments used in the luciferase reporter gene assay. NF-κB binding sites that we analyzed in this study (NF-κB-1286, -1128, -894, -642 and -563) are highlighted in yellow. Predicted transcription start sites have been indicated in the red frame.

pFcRn-1> (-1381)
GCTATAGCTCTGATTCGACCCCTAACCTGGGAACCTCCATGTGCCGTGGGT
NF-κB-1286
GTGGCCCTAAAAAGCAAAGCAAAAAACAAAACAAAACAAAAAAT**GGGAG**
TTTCCATTTCCGCTCGGTGTGTTAAGAACCCGACTGAGGTTCTTATCAGGGA
TGCAAGTTCAATCCCTGGCCTCACTCAGTGGGTTAAGGATCCCGCATTGTT
pFcRn-2> (-1150)
ATGGCTGTGGTATAGGCTGATATCTACAGCTTCAGCTGGACCCGTAGCCT**GG**
NF-κB-1128
GAACTTCCAGATGCCGCATGGCATGTATTGCTGTAAAAAGGAAAAGAACA
AAAAACAAATTGCACACACACGCACGCAAACACACACTCAACTTAGACAC
AGATGTTCACAGCAGCATTATTTACTACAGCCAAAAAGTGAAACGACCCAA
ATGCCCATCAGTTGATTAATGAATCCACAAAATGTGGTTGATCCAGACAATA
NF-κB-894
GAATATTATTCACCCATAGAAAGAAATTA**AGGGAGTTCC**TTGGTGGCTCTGT
GGTTTAAGGATCTGGGATCACTGCTGCGGCTCTGATTACACCTGTGGTTTG
GATTTGATCCCTGGCCCAGGAAATTCTGCACGACCTAGGCGAGGCCAAAA
AAGAAATGAAGTTTTGACACATGGTACCACATGCATAAATCTTGAAAACAG
TATTGGACATGGTGAAAAAAGCCAGACACAAAACGGCACCATATTGTAGGA
NF-κB-642
TTCCATTTCTCTGAAAATCCAGAAGA**GGCAAATTCC**TAGAGACAGAACGTA
pFcRn-3> (-577)
CACATCGGTGATTGCCAGGAGCCGAGGGGAGCAGCGGGGCTACTTAAAGG
NF-κB-563
GGTA**CGGGGTCTCC**TTGGGGATGATGAAAAGGTTCTGGAACTAGGTAGTGG
CCGTGGTGGCACAACAGAGAAGGCGCTAAATGTCACCAACAGGAACGCTT
TTGTTTTGTGCATTTTACCGCAATAAAACGGATGAATGGAAGCATTATAGCG
GGAGTGTGGGCTGCGGGGGTGGGTGGGGGGGTTGGAAGAGGCCCCCAAG
CACGAAGGTATATCAGGACTCTGTTACTGAGGTCCTATCAGAAGCTATCAA
AGTACCGCAGGAGCACTTTAAGGGTACCCTGGGGGAGGGGTTCAAAGGAG
GAAGTGTGGAAGTATCTGGAAGACTTATCACAGCAGGAGCCAGTGAAGAC
pFcRn-4> (-208) **transcription start site-1**
CTGGGGGGGGGTGCTGACGAGGTAAGAAGGGGGCCTGCGCCGATCTCTAA
AGGTGGGGCTCCGATAAGGTTGACTCCCAACGTTGTCATGAATTTGCTGGA

TCGCCGGCACCCTCGAAAAACAAAACAAAACAAAGCCCCCAAACAAAAC

transcription start site-2

TCAAACCAGGGAGCCACAAAGAAAGAGACGGGGAAAAAAAGGACCCA

-1 | EXON-1

AGTTTCTAGAGAGAGGCTGAGACCAGCTCCCGGCTGGGCCCAGGGCCCGG
GGTGTGCCCTGCAGCTGGGGGGCGGGGTTGGATATTCCCGGAGTGGAGGC
GCCTCTCCGGACAGGATGTGAGAGAGGAACTGGGGTCTCCAGTCACGGGA
GCCAGCTGGGGCCGCCGGCGGGAAGGGATCGCGGCTGCTGTGAGCGGGG
CTGGGGCCCCAGAGCCTGGGAGGCGAGGGCCGGGCGCCGGGTTCTCGGCT
CTCCAGGAGGAGGGAGCCCCGGGTCTGGACCTTTGGGTCGGAGGGAGGA
GGGGCGGGCGGGCGGGCGGGCGGGAGCCGAGGACTCGCGGGTCCGAGGG
AGGAGGAGGAGGAGGAGGAGGAGGAGGAGGAGGAGGAGGAGGGGCCGC
CTCCGCCGCCTCCCGGCCTATCCAGACTCTGTCACTCATTAACCCCGACGG
AGCAAACGAAGGGGTGAAAGTTCATCGGGGAGGCGGGCCTTTCGGTCACT
CTCCGGGACAGGAGCCCCGGAGCCCGCCGCCGCAGGGACTGGGGGACGC
AGGGCGGGGCGGGGCGGGGGTCCAGGGAGTCACGCGCCCCCTCCCGCC
CCAGGTCTCCCGCTCAGG

Fig. S2 Alignment of pFcRn and human FcRn upstream sequences of the start codon.

```
                            10        20        30        40        50        60        70        80
                    ....|....|....|....|....|....|....|....|....|....|....|....|....|....|....|....|...
Porcine             GGCTGGGCCCAGGGCCCGGGGTGTGCCCTGCAGCTGGGGGGCGGGGTTGGATATTCCCGGAGT---GGAGGCGCCTCTCCGGA
Human               GGCTGGGCCTAGGGCCTGGGGTGTGCCCTGCAACTGGGGGGTGGGGCTGGATGTTCTTGGAGTCAGGGAGCAGCAGCCTCCCA
Clustal Consensus   ********* ****** *************** ******** **** ***** ***  *****   ****  **  *  *  *

                           110       120
                    ....|....|....|....|....|..
Porcine             ACTGGGGTCTCCAGTCACGGGAGCCAG
Human               ACTGGGGTCTCCAGTCACGGGAGCCAG
Clustal Consensus   ***************************
```
